# Food safety practice and associated factors among food handlers working in food and drinking establishments in Debre Birhan City, North Eastern Ethiopia: A convergent parallel mixed-method study

**DOI:** 10.1371/journal.pone.0346700

**Published:** 2026-04-16

**Authors:** Belachew Tekleyohannes Wogayehu

**Affiliations:** Department of Environmental Health, Debre Birhan Health Science College, Debre Birhan, Ethiopia; Ataturk Universitesi, TÜRKIYE

## Abstract

**Background:**

Food-borne illnesses pose a major global public health threat, affecting up to 30% of people annually in higher-income countries and contributing to hundreds of thousands of deaths each year with the heaviest burden falling on low and middle income countries. In Ethiopia, prior research on food handlers’ safety practices has been limited to quantitative methods offering little insight into cultural, behavioral and institutional barriers. To the best of our knowledge, limited mixed-methods evidence exists in this context. Therefore, this mixed-methods study addresses the gap through providing comprehensive evidence from North Eastern Ethiopia on factors influencing food safety practices.

**Objective:**

This study aims to assess food safety practices and associated factors among food handlers working in food and drinking establishments in Debre Birhan city, North Eastern Ethiopia.

**Methods:**

A convergent-parallel mixed-methods study with a cross-sectional quantitative strand was conducted from January 10 to February 28, 2025, among 415 randomly selected food handlers in Debre Birhan city, North Eastern Ethiopia. Quantitative data were collected using a pre-tested structured questionnaire and observation method and analyzed using STATA version 14. Multivariable binary logistic regression with 95% confidence intervals and a p-value ≤ 0.05 was used to identify factors associated with food safety practices. Qualitative data were analyzed using ATLAS.ti version 8 software.

**Results:**

The magnitude of poor food safety practices among food handlers was 72%. Educational status (AOR = 3.2; 1.206–5.337), working ≤ 2 years (AOR = 2.7; 1.026–4.009), lack of food safety training (AOR = 4.3; 1.997–7.820) and poor knowledge (AOR = 3.9; 1.853–6.983) were statistically associated with poor food safety practice among food handlers. Lack of food safety training, poor risk perception, weak enforcement, lack of standardized guidelines, inadequate facilities and insufficient equipment were identified as barriers for good food safety practice.

**Conclusions:**

Food safety practice among food handlers in the current study was low. Quantitative findings showed that educational status, working ≤ 2 years, lack of food safety training and poor knowledge were factors statistically associated with poor food safety practice. Qualitative findings provided limited access to training, poor risk perception, weak enforcement, lack of standardized guidelines, inadequate equipment and facilities jointly undermine food safety compliance. Addressing these factors particularly through enhanced training and knowledge improvement as well as regular and supportive supervisory, monitoring and evaluation mechanisms could substantially improve adherence to safe food handling and reduce the risk of foodborne diseases.

## Introduction

Food safety is the term used to describe the circumstance and supervision required to ensure the safety of food, wholesomeness and suitability throughout production and consumption. Several nations have made it their top public health priority, which is crucial to preventing foodborne illness and improving human health [1,2]. Since eating is a basic human requirement, access to enough, wholesome food is essential for public health and food security [3,4]. World Health Organization (WHO) defines food safety as the steps and conditions needed to ensure that food is safe, healthy and appropriate for human consumption during production, processing, storage, distribution and preparation [5]. Food safety has drawn more attention worldwide in an effort to safeguard public health and avoid diseases brought on by consuming poor-quality food [5,6].

In developed countries, up to 30% suffer from food-borne illness yearly [7], while developing nations face heightened risks from poor sanitation and inadequate hygiene particularly through transmission routes such as unhygienic food handlers, contaminated surfaces and direct exposure to bacteria or parasites [3]. WHO found that because of poor food safety practices and contaminated food, one in ten individuals worldwide suffer from food-borne illnesses with around 2 million deaths each year [8].

About 70% of diarrheal infections in developing countries link to contaminated food and poor hygiene, driving high morbidity and mortality [5,9]. Urbanization and lifestyle changes have increased dining out, spurring unregulated food establishments with substandard hygiene [10]. In Ethiopia, food handlers’ good safety practices such as personal hygiene, storage practice, cleaning and sanitation and prevention of cross-contamination range from 27.4%–57.1%, varying by factors like water storage availability, experience and hygiene training [3,8,11–13]. In early 2023, Save the Children reported 156 food-borne illness cases (including diarrhea) and 2 fatalities in Somaliland. This suggests that food safety and hygiene procedures may have problem and highlight the significance of assessing food safety practice at Debre Birhan City in which comparable risk may exist [14].

The Ethiopian government has set rules and regulations including licensing requirement and health inspections for the handling of food at public food venues. However, there are obstacles in the way of the country’s successful implementation of food safety regulation. There is little comprehensive understanding of how poor food safety awareness, training gaps, and resource limitations combine to influence food handlers’ compliance and regulatory bodies’ enforcement strategies. A convergent mixed-methods methodology that integrates qualitative insights with quantitative evaluation of capacity and compliance is needed to close this gap. Prior studies conducted in Ethiopia have shown significant variations in food safety practice among food handlers [3,8,11–13]. However, previous studies conducted in Ethiopia [5–7,15] had only used quantitative approaches (rely on numbers) to quantify the extent of the problem so wouldn’t offer up any qualitative insights to change. As a commercial and administrative hub in North Eastern Ethiopia, Debre Birhan attracts a large and diverse population, including residents, travelers and students which heighten the demand for ready-to-eat foods prepared by food handlers of varying levels of training and awareness. Moreover, to the best of the researcher’s knowledge, no study has been conducted to investigate factors associated with poor food safety practice through convergent parallel mixed-method approach. Conducting the research in Debre Birhan was therefore essential to generate context-specific evidence on food safety practices, particularly within the city’s hospitality sector. Such evidence can inform targeted interventions, guide public health policy decisions and strengthen food safety management systems. Ultimately, these efforts contribute to improved public health by protecting consumers and reducing the incidence of food-borne illnesses. In resource constrained areas like Ethiopia where foodborne infections continue to be a major public health concern due to inappropriate handling, a convergent parallel mixed-methods study was crucial as it improves validity, reduces biases such as social desirability and provides richer, more practical recommendations for interventions. Thus, this study would take advantage of the convergent parallel mixed research design to offer sufficient insight into food safety practices and their determinants in food handlers in Debre Birhan.

## Methods and materials

### Ethics statement

This study was conducted in accordance with the Helsinki Declaration. Ethical approval was obtained from the Institutional Ethical Review Committee of Debre Birhan Health Science College (Approval number: ደ/ብ/ጤ/ሳ/ኮ 580/36/2025). Permission letter was obtained from Debre Birhan City Administration Department (No: ኢ/ኤ/አ/ሥ/ቡ/116/2025). Before data collection, written consent was obtained from each study participants. The purpose of the study was explained to all participants and they were assured that their information would not be used for purposes other than scientific research. Participants were informed that participation would be voluntary and that they were free to withdraw at any time without obligation. Confidentiality was maintained by using identification numbers instead of the names of study members.

### Study settings

Debre Birhan is a city in the central Ethiopian highlands at an altitude of 2,840m in North shoa Zone of Amhara Region, about 130 km northeast of Addis Ababa. The city has a total population of 160,408 living in five sub-cities in 2020 G.C; 73,929 were males and 86,479 females. The average annual temperatures during the day and night are 20.7 °C and 8.2 °C. Average annual rainfall is 1219 mm and the mean relative humidity is 31% [16].

### Study design and period

**Fig 1:**
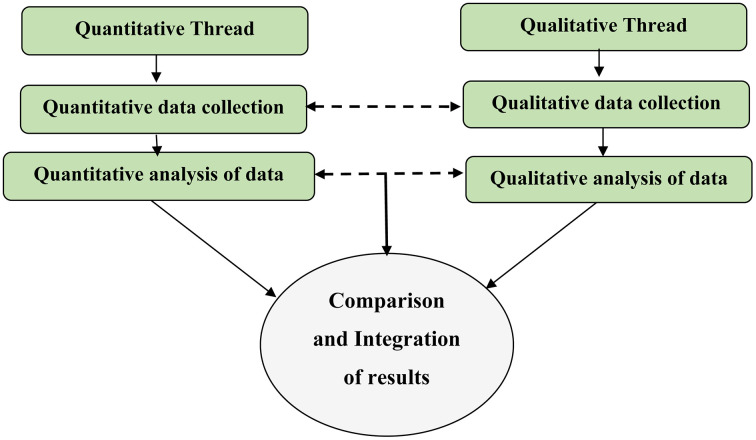
Schematic presentation of convergent parallel mixed study design.

This study employed methodological triangulation using a convergent-parallel mixed-methods design. The study was conducted from January 10 to February 28/2025. Both quantitative and qualitative data were collected concurrently and analyzed independently. Quantitative and qualitative data were integrated by a side by side joint display to cross validate and give explanation of key findings on both sides. The findings were interpreted in an integrated manner within the discussion section, with quantitative and qualitative results presented sequentially to ensure coherence. Quantitative results identifying poor food safety practices among food handlers were complemented by qualitative findings that explored the underlying reasons for these practices. Similarly, statistical associations observed in the survey data were further elucidated through themes emerging from the qualitative interviews, providing deeper contextual understanding (Fig 1).

### Population

Source population for quantitative part: All food handlers working in food and drinking service establishments of Debre Birhan city were the source populations of the current study.

Source population for qualitative part: All food handlers working in food and drinking service establishments and key informants of Debre Birhan city were the source populations of the current study.

### Eligibility criteria

Food handlers working in preparation, cleaning and service areas of food and drinking service establishments were included in this study.

### Sample size determination and sampling procedure

The sample size for this study was computed by using a single population proportion formula by considering the prevalence of good food hygiene practice (p = 43%) from a study conducted in Batu town [15].


$$n=\frac{{\left(\frac{z\alpha }{\text{2}}\right)}^{\text{2}}\;\times P(-P)}{d^{\text{2}}}$$

Where, n = is the required sample size [17]

Z= 95% confidence interval (Zα/2 value =1.96).

P = Prevalence of good food hygiene practice (43%).

d = 5% margin of error.


$$n=\frac{\left({\text{1}.\text{9}\text{6})}^{\text{2}}\times \text{0}.\text{4}\text{3}(\text{1}-\text{0}.\text{4}\text{3}\right)}{({\text{0}.\text{0}\text{5})}^{\text{2}}}\;=\;\frac{\text{3}.\text{8}\text{4}\times \text{0}.\text{2}\text{4}\text{5}\text{1}}{\text{0}.\text{0}\text{0}\text{2}\text{5}}=\;377$$

Finally, by considering 10% of non-response rate, the final sample size for this study was 377 + 38 = 415.

Prior to the start of the study, a census was carried out to find out how many people handled food in all of the food and drink establishments in Debre Birhan city. Across 462 establishments, including 106 hotels, 90 cafeteria-restaurants, 35 cafeterias, 52 juice houses, 94 butcher shops and 85 shuro homes, 1,941 food handlers were found. Participants were selected using a systematic sampling technique. Approximately 4.68, rounded to 5, was the sampling interval (K), which was calculated by dividing the total number of food handlers (1,941) by the intended sample size (415). A random number between 1 and 5 was used to determine the beginning point, which in this case was “3.” Following this, every 5^th^ food handler on the list (i.e., 3^rd^, 8^th^, 13^th^, 18^th^ and so on) was included in the sample until the required sample size of 415 was reached.

To collect qualitative data, participants were selected purposively who may have direct experience or opinions on challenges faced by food handlers’. In addition to this, purposive sampling is particularly effective in qualitative research, where in-depth responses and detailed perspectives are crucial. Moreover, purposive sampling allows the researcher to select participants who are familiar with the local challenges specific to Debre Birhan city. The sample size was estimated on the basis of richness, detailed nature and volume of data collected, relevant published literature, scope of study and the amount of useful information obtained from each participants [18]. In this study, saturation of data was attained when more interviews from in-depth interview and no further new information, themes or insights was obtained from the key informant when response become repetitive and no new codes or categories are identified that was crucial to food safety practice and associated factors. Saturation, the most widely used principle for qualitative sample size determination, was achieved with this large sample due to the heterogeneity of the participants and the broad scope of the study [19]. Proportional numbers of study participants were included from the city. They were interviewed about their food safety experiences and practices. Twelve in-depth interviews and nine key informant interviews would be sufficient to achieve thematic saturation. The key informants were people with knowledge of communicable disease control methods and decision makers on implementation of proper food safety practices in the study institutions and who know the living conditions, beliefs, and behaviors.

### Participant description

The participants in this study comprised two key groups: food and drinking service establishment workers including owners and key informants. Food and drinking service establishment workers were selected using purposive sampling from the city, ensuring diversity in socioeconomic backgrounds, age and gender. A total of 21 individuals participated for twelve in-depth interviews to explore their insights into their experiences and practices regarding food safety practices and nine KI. A total of 9 KI included in which 2 woreda health and health related officers, 2 Environmental Health Officers, 2 Health Extension Workers and 2 owner of food and drinking service establishment and 1 local leader all of whom knowledge of communicable disease control methods and decision makers on implementation of proper food safety practices in the city. These informants provided critical perspectives on the health system’s role and challenges in promoting prolonged food safety practices.

### Outcome and explanatory variables

Outcome variable was:

- Food safety practices (Poor/Good)

Explanatory variables fell into three categories: socio-demographic, institutional and health related and sanitary facility related factors.

**Socio-demographic factors:** Age, gender, education, marital status, work experience and responsibility.

**Institutional and health-related factors**: Food safety training, regular supervision, and routine medical checkups.

**Sanitary facility related factors:** Presence of a refrigerator in the kitchen, suitable water storage equipment, and separate dishwashing systems.

**Other factors**: knowledge of food handlers and attitude of food handlers.

### Operational definitions and outcome measurements

**Food and drinking service establishment:** An establishment that offers breakfast, lunch, dinner, or drinks to big crowds of patrons. These businesses include cafeterias, restaurants, butchers, juice house, shuro house and hotels [20].

**Food safety knowledge (overall):** was measured using 11 item questions containing:

Primary cause of food borne illness, most effective way to prevent food borne illness, time of hand washing by food handlers, time of storage of perishable food, best practice of washing hand before handling food, cross-contamination, acceptable jewelry for food handler, when food handlers sty home, proper way to store raw meat, activity after touching raw meat and how often food contact surface cleaned. Each correct response was scored 1 point and incorrect responses were scored 0.

**Good knowledge:** participants who have scored greater than or equal to the mean value of knowledge measurement questions [21].

**Poor knowledge:** participants who have scored less than the mean value of knowledge measurement questions [21].

**Food safety attitude:** feeling or conviction regarding food safety, as well as the viewpoint of food handlers regarding food safety procedures. In this study, attitude was evaluated using 12 questions. Attitude questions were designed with five point Likert scale [1] strongly disagree, [2] disagree, [3] neutral, [4] agree and [5] strongly agree.

**Positive attitude:** participants who scored greater than the mean score of attitude measurement questions [22].

**Neutral attitude:** participants who scored equal to the mean score of attitude measurement questions [22].

**Negative attitude:** participants who scored less than the mean score of attitude measurement questions [22].

**Food safety practice:** the application of food safety and it was evaluated using structured questionnaires and observing food handlers while serving (working) using an observational checklist. Observational checklist to reduce social desirability bias was used where feasible to complement self-reported data thereby reducing reliance on participant self-report alone. In this study, food safety practice of food handlers were measured using 18 questions and observational checklist. Each correct response was scored 1 point and incorrect responses were scored 0 [23].

**Good practice:** participants who have scored greater than or equal to the mean value of practice measurement questions [23].

**Poor practice:** participants who have scored less than the mean value of practice measurement questions [23].

### Data collection tools and procedures

Data were collected using a structured questionnaire and observational checklist adapted and modified from previously published studies [5,8]. Data were collected in face-to-face interviews using the questionnaire and observation method. The observational component focused on key hygiene and food handling behaviors was used to complement and validate self-reported practices. The questionnaire was structured into five parts; socio-demographic characteristics of study participants with six questions, institutional and health related factors with four questions, sanitary facility related factors with three questions and food safety practices with eight questions, knowledge assessment with eleven questions, attitude assessment with twelve questions and practice assessment with eighteen questions. Before actual data collection, five Environmental Health professionals for data collection and two supervisors were trained by principal investigator for two days to maintain neutrality. In order to maintain anonymity, interviews were held in a private location and participants were guaranteed that their answers would be kept anonymous and would not have an impact on their employment. Food safety practices were assessed using eighteen closed ended questions with two possible answers each: “yes (1)” or “no (0)”. Each response indicating safe practice was assigned a score of one, while responses indicating unsafe practice received a score of zero.

Qualitative data were gathered through semi-structured interviews conducted in a quiet, secure setting to ensure clear recordings with key informants purposively selected based on their roles and academic status. Nine key informant interviews were conducted. The interviews were held in Amharic languages, following a friendly approach by adding probing questions to make the environment easy to participate in and to discuss, apparently. Language barriers were addressed using trained Amharic-speaking interpreters, translated semi-structured interview guides, and questionnaires that included participants’ demographic information to ensure clear, ethical, and effective data collection. Each interview took an average of 15–25 minutes and 20–30 minutes for an in-depth interview and KI respectively. The tool used for each component (i.e., In-depth interviews and KI). Before the actual data collection, pre-test was conducted in nearby Shewarobit town using a sample of 10% with purposively selected participants. Local research assistants enhanced trust and communication, while neutral data collectors avoided interviewing acquaintances to reduce bias. Data collectors received two days of training on consistent, open-ended interviewing, and all interviews were audio-recorded.

### Data quality assurance

To ensure data quality, a structured questionnaire adapted from various previous studies was used. The questionnaire was translated from English to Amharic (the local language commonly spoken in the study area) and then back to English prior to data collection to ensure its consistency and validate the data collection tool. Before the actual data collection, content validity was conducted by Senior Environmental Health professional. The reliability of knowledge, attitude and practice of food safety questionnaires was assessed using a Cronbach alpha test and consistency scores of 0.813, 0.886 and 0.829 respectively. Since the value of Cronbach alpha is greater than 0.7, the reliability in the collected data was good. The questionnaire was verified on a regular basis for completeness and consistency. Incomplete data was recollected again and incomplete data due to withdrawal of study participants from the interview and unwillingness of study participants for the study was considered as non-response. Finally, all the collected data were cleaned to identify and correct outliers or inconsistencies, ensuring accuracy and reliability. Cross-checking was also performed before conducting the analysis.

### Data processing and analysis

Data obtained from the interview on food safety practice were entered into Epidata version 4.6 before being exported into STATA version 14 for data cleaning and descriptive and factor analysis. The data were checked for any missing values and basic quality assurance measures were performed using descriptive statistics results from cross-tabulations (contingency coefficient) and frequency distributions before the statistical analysis. Finally, descriptive statistics were calculated to summarize socio-demographic characteristics of study participants; these consisted of frequency distributions and measures of central tendency and dispersion using tables and figures. The continuous and categorical independent variables were characterized and re-characterized. Analysis of data with respect to factors affecting food safety practice was conducted based on the nature of variables. In this study, knowledge, attitude and practice scores were dichotomized based on mean score to classify participants into “good” and “poor” categories. A bi-variable binary logistic regression model was employed to determine crude odds ratio and 95% CI was used for the association between dependent and independent variables. The variables with P < 0.25 in the bi-variable binary logistic regression were selected as candidate variables and entered into multivariable binary logistic regression to adjust for the possible effect of confounders using a backward stepwise method; Adjusted Odds Ratio (AOR) was determined after adjusting candidate variables in the bi-variable binary logistic regression. Based on the AOR at 95% CI, variables having P ≤ 0.05 were considered statistically significant factors. To test multicollinearity of independent variables, Variance Inflation Factor (VIF) was utilized; all variables had VIF < 5, which means there was no multicollinearity between independent variables. The Hosmer and Lemeshow test was used to check the model’s overall goodness of fit and had p value of 0.42 > 0.05.

### Field notes

Field notes were taken during and after interviews to capture important contextual details. The principal investigator added descriptive observations to supplement recorded and transcribed statements, clarify speaker identity when voices were similar and document nonverbal cues such as body language, pauses, facial expressions and eye contact to aid interpretation.

The author and local translators digitally recorded the interviews, transcribed verbatim and translated from Amharic to English. Data were managed and analyzed using ATLAS.ti version 8 software, employing thematic analysis to identify and interpret patterns in the data [24]. The data were content coded for thematic analysis. Initial coding was based on preset categories developed from the literature and emerging themes were derived from the data [25]. Coded data were analyzed to identify the frequency of key concepts, themes and relationships with textual and structural analyses used to gain a comprehensive understanding of participants’ experiences with food safety practices [18]. Textual analysis refers to the description of what is expressed by the interviewees while structural analysis stands for the interpretation of how it is expressed by the interviewees. In this convergent parallel mixed-method study, inter-rater reliability was evaluated using Cohen’s Kappa coefficient to increase the validity and consistency of the qualitative findings. Two independent researchers coded the interview transcripts using a formal framework based on study objectives and major themes. Inter-coder agreement was assessed with Cohen’s Kappa, accounting for chance agreement. In this study, Cohen’s Kappa coefficient demonstrated substantial agreement (k = 0.76). The coding was reliable, with consistent interpretation of themes on food safety practices among food handlers in Debre Birhan City shown by a high inter-coder agreement score. The study ensured trustworthiness through rigorous procedures addressing credibility, transferability, dependability and confirmability.

### Trustworthiness of the study

**Member checking:** The transcribed data, interpretations, and conclusions were returned to the contestants to correct errors and challenges that were professed as wrong interpretations and data triangulation using multiple respondents with different age groups.

**Prolonged engagement:** The interview took an average of twenty minutes to thirty minutes with active participation. Therefore, the study was conducted on food safety practice of food handlers who work in food and drink service establishments.

**Credibility:** The results, all In-depth interviews and KI, depending on guidelines, were evaluated by experts before the data collection.

**Transferability:** To uphold the transferability of the finding, appropriate probes were used to obtain comprehensive information during In-depth interviews and KI, as well as field notes and digital audio records, were used for all interviews before and during the analysis.

**Dependability:** To maintain the trustworthiness of the research process, member checking and prolonged engagements were made after In-depth interviews and KI. Conformability of this study was assured by persistent observations of participants’ body movements, facial expressions, eye gaze and tone of speech, and everything that was recorded at the time of the interview. Peer debriefing was made by experts. Flexibility and a friendly relationship between researchers and participants were mandatory to receive input and comments.

## Results

### Socio-demographic characteristics of food handlers

From a total of 415 study participants recruited for this study, 405 participants provided complete information with response rate of 97.6%. Most participants (77.5%) were aged 20–29 and overwhelming majorities (88.6%) were female. Additionally, around 299 participants (73.8%) had worked as food handlers for two years or less (Table 1).

**Table 1:** Socio-demographic characteristics of food handlers in Debre Birhan city, North Eastern Ethiopia, 2025 (n = 405).

Variables	Frequency (n)	Percentage (%)
**Age of the respondent**		
20-29	314	77.5%
≥ 30	91	22.5%
**Sex**		
Male	46	11.4%
Female	359	88.6%
**Marital status**		
Single	241	59.5%
Married	117	28.9%
Divorced	47	11.6%
**Educational level**		
Primary school	147	36.3%
Secondary school	137	33.8%
College and above	121	29.9%
**Work experience**		
≤ 2 years	299	73.8%
> 2 years	106	26.2%
**Responsibility**		
Main Chef/cook	157	38.8%
Assistant Chef/cook	137	33.8%
Waiter	111	27.4%

### Institutional and health related factors of food handlers

The vast majority of food handlers, 280 (69.1%), had no training on food safety practices. Of the total food handlers, 180 (44.4%) reported no regular supervision on food safety practices. More over 178 (43.9%) of the study participants did not get regular medical checkups (Table 2).

**Table 2:** Institutional and health related factors of food handlers in Debre Birhan city, North Eastern Ethiopia, 2025 (n = 405).

Variables	Frequency (n)	Percentage (%)
Food safety training		
No	280	69.1%
Yes	125	30.9%
Regular supervision/Sanitary inspection		
No	180	44.4%
Yes	225	55.6%
Regular medical checkup/ever had medical checkup		
No	178	43.9%
Yes	227	56.1%

### Sanitary facility related factors of food handlers

In this study, majority 314 (77.5%), did not use a refrigerator in the kitchen. In terms of suitable water storage equipment, around 174 (42.9%) reported having appropriate water storage equipment, while 227 (56.1%) utilized separate dishwashing systems (Table 3).

**Table 3:** Sanitary facility related factors of food handlers in Debre Birhan city, North Eastern Ethiopia 2025 (n = 405).

Variables	Frequency (n)	Percentage (%)
Refrigerator in the kitchen		
No	91	22.5%
Yes	314	77.5%
Availability of appropriate water storage equipment		
No	231	57.1%
Yes	174	42.9%
Use separate dishwashing systems		
No	178	43.9%
Yes	227	56.1%

### Knowledge of food handlers towards food safety practice

**Fig 2:**
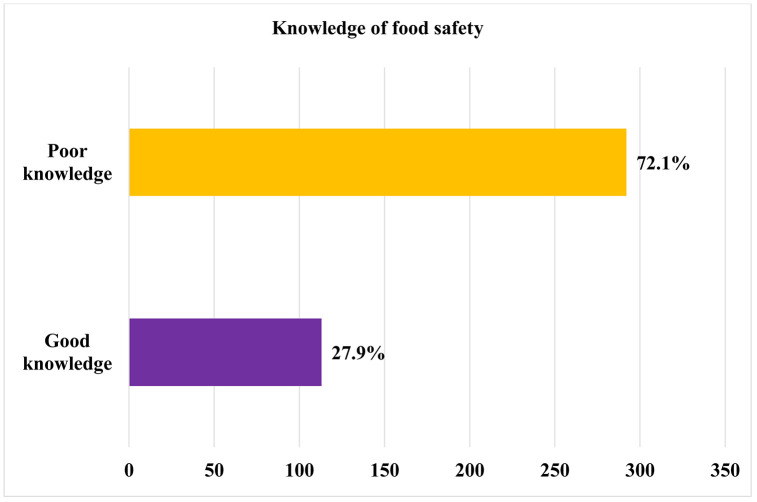
Schematic illustration showing the level of knowledge of food handlers regarding food safety practices in food and drinking establishments in Debre Birhan City, North Eastern Ethiopia, 2025 (n = 405).

In this study, the mean good knowledge of food handlers towards food safety practice was 113 (27.9%) and the mean poor knowledge of food handlers towards food safety practice was 292 (72.1%). The findings of this study showed that 277 (68.4%) of food handlers had good knowledge in which poor personal hygiene is the primary cause of food borne illness (Table 4 and Fig 2).

**Table 4:** Knowledge of food handlers towards food safety practice in Debre Birhan city, North Eastern Ethiopia, 2025 (n = 405).

S/N	Questions	Correct Response	Frequency (n)	Percentage (%)
1	Primary cause of food borne illnesses	Poor personal hygiene	277	68.4%
2	Most effective way to prevent food borne illness?	Practicing good hygiene	39	9.6%
3	Recommended hand washing duration	At least 20 seconds	23	5.7%
4	Safe time for perishable foods at room temperature	2 hours	117	28.9%
5	Best practice for washing hands before handling food	Wash with soap and warm water for at least 20 seconds	82	20.2%
6	Meaning of Cross-contamination	Transfer of bacteria/other contaminants	64	15.8%
7	Acceptable jewelry for food handlers?	A plain wedding band	39	9.6%
8	When should food handlers stay home	When experiencing vomiting or diarrhea	50	12.3%
9	Proper way to store raw meat	On the bottom shelf to prevent dripping onto other foods	95	23.5%
10	What to do after touching raw meat	Wash hands thoroughly	37	9.2%
11	Cleaning frequency of food-contact surfaces	Every 4 hrs during continuous use	53	13.1%
	**Mean knowledge status (n, %)**	**Good knowledge**	**113**	**27.9%**
		**Poor knowledge**	**292**	**72.1%**

### Attitude of food handlers towards food safety

The result of the current study revealed that 104 (25.7%) of food handlers hand positive attitude towards food safety practice, 109 (26.9%) of food handlers had neutral attitude and 192 (47.4%) of food handlers had negative attitude towards food safety practice (Table 5).

**Table 5:** Attitude of food handlers towards food safety practice in Debre Birhan city, North Eastern Ethiopia, 2025 (n = 405) Ethiopia, 2025 (n = 405).

S/N	Assessment questions	Strongly Agree (5) (n, %)	Agree (4) (n, %)	Neutral (3) (n, %)	Disagree (2) (n, %)	Strongly disagree (1) (n, %)
1	Personal hygiene is essential in preventing foodborne illnesses	193 (47.7%)	45 (11.1%)	167 (41.2%)	----	----
2	Following food safety rules helps prevent foodborne illnesses	38 (9.4%)	30 (7.4%)	126 (31.1%)	143 (35.3%)	68 (16.8%)
3	I feel responsible for maintaining food safety in my workplace	72 (17.8%)	62 (15.3%)	105 (25.9%)	166 (41%)	*----*
4	I always follow hand-washing procedures, even when I am busy	23 (5.7%)	41 (10.1%)	90 (22.2%)	129 (31.9%)	122 (30.1%)
5	Wearing gloves while handling ready-to-eat food is necessary	20 (4.9%)	18 (4.4%)	101 (24.9%)	176 (43.5%)	90 (22.2%)
6	I believe it is important to report coworkers who do not follow food safety rules	31 (7.7%)	39 (9.6%)	77 (19%)	137 (33.8%)	121 (29.9%)
7	I feel comfortable reminding coworkers about food safety practices	48 (11.9%)	52 (12.8%)	126 (31.1%)	160 (39.5%)	19 (4.7%)
8	If I feel slightly sick, I should still avoid handling food	26 (6.4%)	39 (9.6%)	112 (27.7%)	129 (31.9%)	99 (24.5%)
9	Food safety training is beneficial for my job	221 (54.6%)	23 (5.7%)	101 (24.9%)	60 (14.8%)	*----*
10	I am committed to following food safety procedures at all times	21 (5.2%)	15 (3.7%)	128 (31.6%)	131 (32.3%)	110 (27.2%)
11	Thoroughly washing chopping board prevent cross contamination	99 (24.4%)	20 (4.9%)	125 (30.9%)	141 (34.8%)	20 (4.9%)
12	We have to ensure internal temperature of food are checked before use	38 (9.4%)	29 (7.2%)	55 (13.6%)	274 (67.7%)	9 (2.2%)
	**Overall attitude status (n, %)**	**Positive attitude**				**104 (25.7%)**
		**Neutral**				**109 (26.9%)**
		**Negative attitude**				**192 (47.4%)**

### Practice of food handlers towards food safety

**Fig 3:**
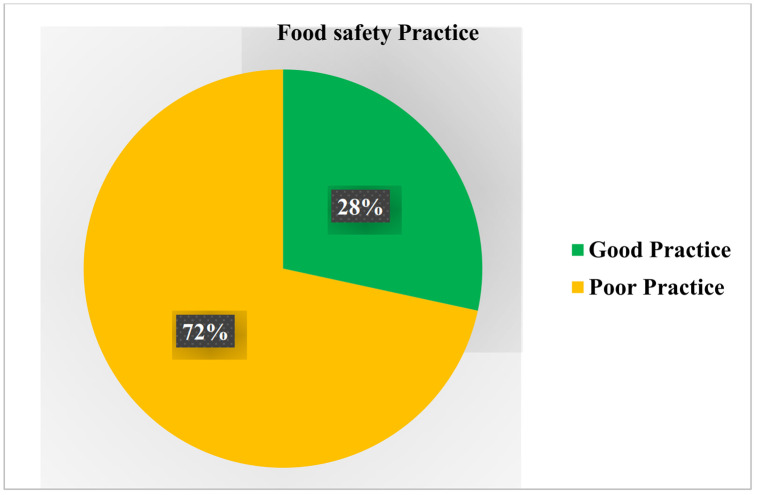
Schematic illustration showing the level of food safety practices among food handlers working in food and drinking establishments in Debre Birhan City, North Eastern Ethiopia, 2025 (n = 405).

In this study**,** the magnitude of good food safety practice of food handlers was 115 (28%) with 290 (72%) of food handlers had poor food safety practice. Additionally, 179 (44.2%) of food handlers had purchased food inputs from the legal market or reputable suppliers and 285 (70.4%) of food handlers wear a work cloth uniform or apron while working (Table 6 and Figure 3).

**Table 6:** Practice of food handlers towards food safety in Debre Birhan city, North Eastern Ethiopia, 2025 (n = 405).

S/N	Variables		Good Practice (Yes) n (%)	Poor Practice (No) n (%)
1	Had purchased food inputs from the legal market or reputable suppliers		179 (44.2%)	226 (55.8%)
2	Slaughter premises for all slaughtered animals		161 (39.8%)	244 (60.2%)
3	Ensured the health status of all slaughtered animals by an authorized body		79 (19.5%)	326 (80.5%)
4	Food handlers did not handle or process any food or meat when they had injuries to their hands		151 (37.3%)	254 (62.7%)
5	Food handlers wear face masks or shields in public food establishments		51 (12.6%)	354 (87.5%)
6	Food handlers reported washing their hands with soap for at least 20 s at key moments during work time		126 (31.1%)	279 (68.9%)
7	Food handlers reported regularly trimming their nail and had seen the trimmed beard		102 (25.2%)	303 (74.8%)
8	Food handlers wear a cap or head covering while selling meat, cooking, or serving food		154 (38%)	251 (62%)
9	Food handlers wear a work cloth uniform or apron while working		285 (70.4%)	120 (29.6%)
10	Food handlers had removed jewelers (hand and ring jewelers) while butchering, serving, or cooking food		33 (8.1%)	372 (91.9%)
11	Food handlers use a thermometer to measure cooked food’s internal temperature		7 (1.7%)	398 (98.3%)
12	Food establishments use refrigerator temperature monitors or tag		46 (11.4%)	359 (88.6%)
13	Food handlers reported always cleaning and sanitizing areas of food establishments (kitchen, a place where meat is sold and stored)		141 (34.8%)	264 (65.2%)
14	Food handlers reported double-checking the expiration dates of packed food input		136 (33.6%)	269 (66.4%)
15	Food handlers never washed raw meats in the sink before cooking		361(89.1%)	44 (10.9%)
16	Food handlers reported discarding unsafe foods held at 40°F (9°C) for more than 2 h		19 (4.7%)	386 (95.3%)
17	Food handlers reported food establishment managers monitor workers for food safety practices		25 (6.2%)	380 (93.8%)
18	Public food establishments were supervised by authority bodies for their food safety practices		14 (3.5%)	391 (96.5%)
	**Mean response (n = 405)**	**Good Practice**	**115 (28%)**	
		**Poor Practice**	**290 (72%)**	

### Factors associated with food safety practice among food handlers

In the bivariate logistic regression analysis, factors like educational status, work experience, food safety training, water storage equipment, regular medical checkup, regular supervision, separate dishwashing systems and knowledge on food safety were candidate variables of food safety practices at p < 0.25. Multivariable analysis (quantitative findings) indicated that, educational status, work experience, food safety training and knowledge on food safety were significantly associated with food safety practice at p ≤ 0.05. In this study, quantitative findings showed that food handlers who completed primary education were 3.2 times more likely to have poor food safety practice than food handlers who completed college and above. Qualitative findings corroborated this (poor risk perception) with participants frequently undervalue the occurrence and severity of food-borne illnesses. Again, quantitative findings showed, food handlers who had worked for ≤ 2 years were 2.7 times more likely to have poor food safety practice as compared to food handlers who had worked for > 2 years. Qualitative findings supported this with participants describing poor risk perception of the impact of poor food safety practice Table 7).

**Table 7:** Bivariate and multivariate logistic regression analysis of factors associated with food safety practice among food handlers in food establishments in Debre Birhan city, North Eastern Ethiopia, 2025 (n = 405).

Variables		Practice		COR (95% CI)	AOR (95% CI)	P-value
**Poor**	**Good**
Educational status	Primary school	108	39	5.02 (2.147-10.36)	3.2 (1.206-5.337)	**< 0.001**
	Secondary school	92	45	3.71 (1.426-4.963)	0.78 (0.496-1.881)	0.072
	College and above	43	78	1	1	
Work experience	≤ 2 years	233	66	5.6 (1.313-8.839)	2.7 (1.026-4.009)	**< 0.001**
	> 2 years	41	65	1	1	
Food safety training	No	246	34	6.1 (2.378-12.36)	4.3 (1.997-7.820)	**< 0.001**
	Yes	68	57	1	1	
Water storage equipment	No	170	61	1.29 (1.113-3.884)	0.602 (0.364-1.996)	0.088
	Yes	119	55	1	1	
Regular medical checkup	No	80	98	2.22 (1.55-4.381)	0.76 (0.115-1.326)	0.061
	Yes	61	166	1	1	
Regular supervision	No	117	63	1.24 (1.037-3.805)	0.314 (0.028-2.783)	0.081
	Yes	135	90	1	1	
Separate dishwashing systems	No	122	56	1.16 (1.09-3.221)	0.56 (0.16-1.1)	
	Yes	151	76	1	1	0.113
Knowledge on food safety	Poor	201	91	4.36 (2.881-9.930)	3.9 (1.853-6.983)	**< 0.001**
	Good	38	75	1	1	

*AOR = Adjusted Odds Ratio and COR = Crude Odds Ratio*

### Theme and sub-theme and categories

Out of twelve in-depth interviewees, six interviewees practice good food safety practice and the remaining six interviewees practice poor food safety practice during the study period. In this study, thematic saturation across both participant groups (i.e., in-depth interviews and key informants) was achieved by the 10^th^ in-depth interview and the 6^th^ key informant interview. Completion of all twelve in-depth interviews and 9 key informants allowed for confirmatory saturation and strengthened the credibility of the qualitative findings. The analysis was focused on the study grand theme, sub-themes, and each of these sub-themes was analyzed in detail with its corresponding categories. The respondents saying on each part were carefully separated and written in the appropriate ways of qualitative research writing (Table 8).

**Table 8:** Themes and sub-themes and categories for exploring barriers for food safety practice among food handlers in Debre Birhan city, North Eastern Ethiopia, 2025 (n = 405).

Grand theme	Sub-themes	Categories
Barriers for good food safety practice	Lack of food safety training	• Limited training for food handlers • Misconceptions about hygiene
	Poor risk perception	• Underestimation of foodborne illnesses • Lack of awareness about contamination sources
	Weak enforcement	• Inconsistent inspections • Limited implementation of food safety laws
	Lack of standardized guidelines	• Informal food businesses • Absence of proper food safety protocols
	Inadequate facilities	• Limited access to clean water • Poor waste disposal systems
	Insufficient equipment	• Shortage of personal protective equipment (gloves, aprons)

### Barriers to good food safety practice

This section presents the key barriers to good food safety practice identified in the study, categorized into distinct themes and subthemes.

### Lack of food safety training

In Debre Birhan city, one of the biggest problems facing food handlers is the absence of official food safety training. Numerous employees report gaining knowledge through unofficial means like observation or prior experiences, which might not be enough to guarantee appropriate food safety procedures. Moreover, in Debre Birhan city, food workers frequently have misconceptions regarding proper sanitary procedures. Without the right training, employees may use antiquated or inaccurate techniques to guarantee the safety of food. Misconceptions such as the belief that food is safe if it smells or looks good can cause contamination and raise the risk of foodborne illnesses. The subjects emphasized the importance of food safety training in good food safety practice. One participant noted that:

*“Regular food safety training would be beneficial, in my opinion, particularly when handling perishable goods and raw meat. It’s not only about the tools; it’s also about understanding what to do at each step. Though we don’t have enough time to learn, there are moments when I question if I am following the correct methods. There hasn’t been any explicit instruction, so it’s challenging to keep up with what I don’t know.”*

### Poor risk perception

In Debre Birhan city, food handlers frequently undervalue the occurrence and severity of food-borne illnesses. Inadequate food safety procedures could result from this misconception, raising the possibility of contamination and disease. Cross-contamination can happen via dirty utensils, dish cloths, or even incorrect storage, yet many food handlers in Debre Birhan city are not aware of this. Due to this knowledge gap, there is a greater chance of contamination during food safety procedures and inadequate food safety procedures. The subjects emphasized poor risk perception among food handlers was a great challenge in implementing good food safety practice. One participant noted that:

*“Previously, I believed food poisoning to be an uncommon occurrence that might only occur during large outbreaks. I was unaware of its prevalence and how it may occur in communities as tiny as ours. Moreover, I had believed that consuming bad food was the only way to contract a foodborne illness. I was unaware of issues like improper hand washing and bacteria from cross-contamination.” (Male, 34 years old, chef of food and drink service establishment at Debre Birhan city, kebele 05)*.

*“The ignorance of food handlers regarding the origins of contamination is a significant barrier to food safety. A lack of knowledge about how food becomes infected causes incorrect handling and storage. Hand washing and segregating raw and cooked meals are two basic hygiene habits that are frequently disregarded because of habit or false information. There is still a knowledge gap and hazardous habits persist in the absence of continuous training, despite our best efforts. Food workers need more hands-on training and educational programs to help them recognize contamination risks and adopt safer practices.” (Female, 30 years old, Health Extension Worker at Debre Birhan city health center, kebele 04).*

### Weak enforcement

Infrequent inspections might give food handlers the impression that their actions are not held accountable, which can result in uneven compliance with food safety laws. One of the biggest concerns for food handlers is the failure to enforce of food safety regulations. Food handlers might believe that observing the regulations is unnecessary. The subjects’ emphasized weak enforcement on implementing food safety regulations was another barrier to practicing good food safety. One participant said that:

*“Food safety must be guaranteed, but there are many barriers to overcome. The inability to do routine and comprehensive inspections of every food outlet due to resource limitations is a significant problem. Budget and staffing constraints hinder our capacity to properly enforce regulations.” (Male, 38 years old, health and health related officer at Debre Birhan city administration health bureau, kebele 04).*

### Lack of standardized guidelines

Because of their nature, Debre Birhan city’s informal food companies frequently lack standardized regulations. Food safety can be dangerous without established protocols, raising the possibility of contamination or foodborne illnesses. Food handlers could not be aware of important practices in the absence of written instructions or explicit procedures, which could result in unsafe behaviors and possible food contamination. The subjects’ also emphasized lack of standard guidelines was also another challenge in practicing good food safety. One participant said that:

*“The lack of precise and uniform food safety guidelines is one of the main difficulties to guaranteeing food safety. Many food enterprises function without the necessary regulations, which results in uneven hygienic standards. Despite the existence of general food safety laws, there aren’t enough specific, legally binding procedures that are adapted to regional circumstances.” (Female, 33 years old hygiene and environmental health officer at Debre Birhan city administration health bureau, kebele 04).*

*“Although the food appears to be well-prepared and stored in the back, I don’t see much attention to detail when it is presented. I have never seen anyone use thermometers or check food temperatures. I had observed meals being served at the incorrect temperature on occasion.” (Male, 44 years old, customer of food and drink service establishment at Debre Birhan city, kebele 01).*

### Inadequate facilities

Food handlers find it difficult to follow basic hygiene procedures, such frequent hand washing and sanitizing kitchenware, in the absence of a dependable and secure water supply. Practice hand washing without basic hygiene procedures increased risk of foodborne infections. Ineffective waste management techniques raise the possibility of contamination. The subjects’ also emphasized lack of adequate facilities in food and drink service establishments was another challenge in practicing good food safety. One participant said that:

*“Despite numerous challenges we encounter on a daily basis, we take food safety very seriously. Access to safe drinking water and adequate sanitation is one of the main issues; without these, hygiene maintenance becomes extremely challenging. Sometimes we lack the funds to purchase cleaning tools, gloves, or even appropriate storage containers.” (Female, 34 years old, food handler in food and drink service establishment at Debre Birhan city, kebele 07).*

*“Here, we lack a suitable mechanism for disposing of waste. The trash accumulates, and occasionally we are forced to dispose of it on the street, which compromises the safety of the food.” (Female, 37 years old, owner of food and drink service establishment at Debre Birhan city, kebele 02).*

### Insufficient equipment

In Debre Birhan city, inadequate equipment poses a serious threat to food safety. Food handlers express worry and anxiety about whether the food is safe to serve. Foodborne illness risk may rise as a direct result of this. A major obstacle to safe food safety techniques is the lack of personal protective equipment such as aprons and gloves. Workers express a sense of powerlessness when they lack the equipment they need to perform their jobs effectively, and this deficit appears to be a persistent issue. The subjects’ also emphasized lack of adequate equipment in food and drink service establishments was another challenge in practicing good food safety. One participant said that: *“A basic requirement for handling food properly is personal protective clothing, such as aprons and gloves. Although there is little we can do about it, I believe that the risk of contamination rises during shortages. Sometimes we are forced to touch food with our bare hands because we frequently run out of gloves. It’s not ideal, but we have no other option if we don’t have enough gloves. I am aware of the need of aprons, but we don’t always have enough, and when we do, they are worn out and in poor shape. Wearing them would increase my confidence in the safety of food.” (Female, 32 years old, food handler in food and drink service establishment at Debre Birhan city, kebele 08).*

## Discussion

The finding of this study revealed that, the overall magnitude of poor food safety practice among food handlers of Debre Birhan city was 72%. The finding was in agreement with the study conducted in Ethiopia (79.1%) [22], Addis Ababa, Ethiopia (72.6%) [26] and (69.2%) [27]. Moreover, the result of the current study was higher in comparison with studies conducted in Ethiopia (51%) [28], Mettu and Bedelle town, Ethiopia (44.9%) [8], Sub-Saharan African countries (49.32%) [29], Iran (70.7%) [30], Kerman (36.9%) [31], Nigeria (60%) [32], North Central Nigeria (7.9%) [33], Ghana (44.2%) [34] and Sudan (69.5%) [35]. The difference might be because of the disparity in study setting, large sample size. The disparity might also be due to poor knowledge (72.1%), completed primary education (36.3%), lack of food safety training (69.1%), absence of regular supervision (57%), poor risk perception, lack of standardized guidelines and insufficient equipment in the present study. Moreover, the difference might also be due to the variation in attitude of food handlers in which 47.4% of food handlers had negative attitude and also weak enforcement and inadequate facilities in the current study.

The finding of the current study was relatively lower in comparison with studies conducted in Bangladesh (83.7%) [36] and (83.3%) [37]. The disparity might be due to the difference in food safety knowledge among food handlers in which only 20% of food handlers had good food safety knowledge in Bangladesh [36] whereas about 27.9% of food handlers had good food safety knowledge in the present study. In addition, the variation might also be due to the difference in food safety attitude of food handlers where only 18.6% of food handlers had positive attitude in Bangladesh [37] and about 25.7% of food handlers had positive attitude in the current study. Again the disparity might be due to educational status difference where 61% of food handler completed primary education and below [36] whereas only 36.3% of food handler completed primary education in the current study. Moreover, food safety training might also cause the observed disparity where 94.7% had no received training whereas only 69.1% hand no food safety training in the current study.

Quantitative findings of the current study showed that food handlers who completed primary education were 3.2 times more likely to have poor food safety practice than food handlers who completed college and above. Qualitative findings corroborated this (poor risk perception) with participants frequently undervalue the occurrence and severity of food-borne illnesses. This finding was supported with other studies conducted in Ghana [38], Kenya [39] and Ethiopia [40] and in which food handlers with low educational status were 3.42, 2.57 and 4 times more likely to have poor food safety practices than those who completed higher education respectively. The finding was also supported with study conducted in Debarq, Ethiopia [41] and Uganda [42]. This is due to the fact that people with higher education frequently encounter more regimented training and learning settings, which enhances their health literacy and awareness. Individuals with only an elementary education, on the other hand, could not have the abilities and information necessary to apply food safety regulations in an efficient manner, which could lead to their participating in risky behaviors.

Again, quantitative findings of the present study showed that food handlers who had worked for ≤ 2 years were 2.7 times more likely to have poor food safety practice as compared to food handlers who had worked for > 2 years. Qualitative findings supported this with participants describing poor risk perception of the impact of poor food safety practice. The finding was supported with other studies conducted in Ethiopia [13] and [43] in which food handlers with who had low work experience were 2.85 and 1.95 times more likely to have poor food safety practice than those who had longer work experience. This may be because they haven’t been exposed to the different conditions and difficulties that come up when handling food. Furthermore, less experienced food handlers might not be able to solve problems on their own and might be more dependent on peers or superiors for direction.

Quantitative findings of the current study showed that food safety training was also significantly associated with food handler’s food safety practice. In this study, food handlers who had no received food safety training were 4.3 times more likely to have poor food safety practice than food handlers who receive food safety training. Qualitative findings verified this with participants describing limited access to formal training, lack of standardized guidelines and lack of refresher sessions. The result of the current study was supported by studies conducted in Bangladesh [36], Ghana [38], Bishoftu, Ethiopia [44], Gondar, Ethiopia [28] in which food handlers who did not take food safety training were 8.98, 5.97, 4.8 and 4.01 times more likely to have poor food safety practice than those who take food safety training respectively. These patterns may be due to the effectiveness of occupational health and safety training, specifically the provision of pertinent information about the impacts of poor food safety and the ability of personal protective equipment to prevent food contamination, on the behavior of workers. Moreover, quantitative findings of the current study showed that food handlers who had poor knowledge towards food safety practice were 3.9 times more likely to have poor food safety practice than food handlers who had good knowledge. Qualitative findings substantiated this with participants describing poor risk perception of the impact of poor food safety practice. The findings was supported with different studies conducted in Ethiopia [10,45–47] and [23] in which food handlers with poor knowledge on food safety were 2.99, 2.11, 2.31, 3.04 and 3.84 times more likely to have poor food safety practice than those food handlers who had good food safety practice respectively. The reason for this might be due to the fact that food handlers that are poor knowledgeable about safe handling practices, storage methods, and hygiene increase the chance of contamination from things like raw and cooked food coming into contact with one another or from incorrect storage at dangerous temperatures. A lack of knowledge about personal hygiene practices, such as frequent hand washing and wearing clean clothes, makes it more likely that dangerous pathogens will be spread. The qualitative input has provided a context for the explanation of the low adherence patterns, particularly in respect of weak institutional enforcement, lack of standardized guidelines and lack of perception of risk.

### Limitations of the study

This study has the following limitations. Since the study was a cross-sectional study, it doesn’t establish a cause-effect relationship and may be influenced by social desirability and recall biases. The absence of design-effect adjustment or multilevel/cluster-robust analysis and the mean-based dichotomization of KAP scores represent key methodological limitations of this study. The inability to conduct laboratory investigation to check the safety of food and utensils in food and drink service establishments due to financial constraint was described as the limitation of this study. Additionally, the inability to address potential intra-cluster correlation among food handlers with in the same establishments was also described as the limitation of this study.

## Conclusions and recommendations

Food safety practice among food handlers in the current study was low. Quantitative findings revealed that educational status, working ≤ 2 years, lack of food safety training and poor knowledge were factors statistically associated with poor food safety practice. Qualitative findings provided limited access to training, poor risk perception, weak enforcement, lack of standardized guidelines, inadequate equipment and inadequate facilities jointly undermine food safety compliance. It is necessary to improve in-service training as one of the key areas for intervention to improve direct knowledge and skills of food handlers on food safety, improve risk perception and clarify the importance of standardized guidelines in workplace to contribute for the success of SDG 3 (good health and well-being). Future research must employ validated knowledge, attitude and practice scales and longitudinal designs to monitor change in behaviors.

## Supporting information

S1 File Structrued questionnaires to assess ‘Food Safety Practice and Associated Factors among Food Handlers Working in Food and Drinking Establishments in Debre Birhan city, North Eastern Ethiopia: A Mixed-Method Study’. (DOCX)

S2 File Data set that contains data generated for the study titled “Food Safety Practice and Associated Factors among Food Handlers Working in Food and Drinking Establishments in Debre Birhan City, North Eastern Ethiopia: A Convergent Parallel Mixed-Method Study.” (XLSX)

## Abbreviations

AOR: Adjusted Odds Ratio
KI: Key Informant
WHO: World Health Organizations
