# Supplementary material for: Food safety practice and associated factors among food handlers working in food and drinking establishments in Debre Birhan City, North Eastern Ethiopia: A convergent parallel mixed-method study
Source: PLoS One. 2026 Apr 16;21(4):e0346700. doi: 10.1371/journal.pone.0346700 (PMC13086347; doi:10.1371/journal.pone.0346700)
Supplement: S1 File — (DOCX) [file pone.0346700.s001.docx]

## **Questionnaire (English version)**

Debre Birhan Health Science College, Department of Environmental Health: A questionnaire designed to assess ‘Food Safety Practice and Associated Factors among Food Handlers Working in Food and Drinking Establishments in Debre Birhan city, North Eastern Ethiopia: A Mixed-Method Study’

Date of data collection __________________

1. Questionnaire code _________________
2. Name of Sub-city __________________
3. Interviewer Name __________________
4. Supervisors Name _________________
5. Status of the interview: Completed Partially completed

Please encircle the correct answer and write a correct number in the space provided, example age in years using pen

| **Part I: Socio-Demographic characteristics (100)** | | | | | |
| --- | --- | --- | --- | --- | --- |
| ***Q.No*** | ***Questions*** | ***Responses/units*** | ***Coding/values*** | | ***Remark*** |
| 101 | Sex (study participant) | Female | 0 | |  |
|  |  | Male | 1 | |  |
| 102 | What is your age? (study participant) | years |  | |  |
| 104 | What is your marital status? (study participant) | Single | 0 | |  |
|  |  | Married | 1 | |  |
|  |  | Divorced | 2 | |  |
|  |  | Widowed | 3 | |  |
| 105 | What is your educational status? (study participant) | Cannot able to read and write | 0 | |  |
|  |  | Primary education | 1 | |  |
|  |  | Secondary education | 2 | |  |
|  |  | College diploma and above | 3 | |  |
| 106 | What is your work experience (years) | Years |  | |  |
| 107 | What is your main responsibility? | . |  | |  |
| **Part II: Institutional and health related factors of food handlers and Sanitary facility related factors (200)** | | | | | |
| ***Q.No*** | ***Questions*** | | ***Coding/***  ***values*** | | **Remark** |
|  |  |  | **No** | **Yes** |  |
| 201 | 1. Do you take food safety training? | | 0 | 1 |  |
| 202 | 1. Is there regular supervision/sanitary inspection by concerned body? | | 0 | 1 |  |
| 203 | 1. Do you regularly apply medical check-up/ever had medical check-up | | 0 | 1 |  |
| 204 | 1. Do you have refrigerator in the kitchen? | | 0 | 1 |  |
| 205 | 1. Is there availability of appropriate water storage equipment? | | 0 | 1 |  |
| 206 | 1. Do you utilize separate dishwashing systems while working? | | 0 | 1 |  |
|  | **Part III:** **Knowledge of food handlers towards food safety practice** | |  |  |  |
| 207 | What is the primary cause of foodborne illnesses? | |  |  |  |
| 208 | 1. Poor personal hygiene | | 0 | 1 |  |
| 209 | 1. Overcooking food | | 0 | 1 |  |
| 210 | 1. Using too much salt | | 0 | 1 |  |
| 211 | 1. Eating too many carbohydrates | | 0 | 1 |  |
| 212 | What is the most effective way to prevent foodborne illness? | |  |  |  |
| 213 | 1. Cooking food longer | | 0 | 1 |  |
| 214 | 1. Practicing good hygiene | | 0 | 1 |  |
| 215 | 1. Using extra seasoning | | 0 | 1 |  |
| 216 | 1. Storing food in any container | | 0 | 1 |  |
| 217 | How long should food handlers wash their hands? | |  |  |  |
| 218 | 1. 5 seconds | | 0 | 1 |  |
| 219 | 1. 10 seconds | | 0 | 1 |  |
| 220 | 1. 15 seconds | | 0 | 1 |  |
| 221 | 1. At least 20 seconds | | 0 | 1 |  |
| 221 | How long can perishable foods safely sit at room temperature before they should be refrigerated or discarded? | |  |  |  |
| 222 | 1. 1 hour | | 0 | 1 |  |
| 223 | 1. 2 hours | | 0 | 1 |  |
| 224 | 1. 4 hours | | 0 | 1 |  |
| 225 | 1. 6 hours | | 0 | 1 |  |
| 226 | What is the best practice for washing hands before handling food? | |  |  |  |
| 227 | 1. Rinse with cold water | | 0 | 1 |  |
| 228 | 1. Wash with soap and warm water for at least 20 seconds | | 0 | 1 |  |
| 229 | 1. Use hand sanitizer only | | 0 | 1 |  |
| 230 | 1. Wipe hands with a towel | | 0 | 1 |  |
| 231 | What is cross-contamination? | |  |  |  |
| 231 | 1. Cooking food for too long | | 0 | 1 |  |
| 233 | 1. Storing food in the wrong container | | 0 | 1 |  |
| 234 | 1. The transfer of bacteria or other contaminants from one surface to another | | 0 | 1 |  |
| 235 | 1. Washing food with hot water | | 0 | 1 |  |
| 236 | Which type of jewelry is acceptable for food handlers to wear while working? | |  |  |  |
| 237 | 1. Rings with stones | | 0 | 1 |  |
| 238 | 1. Bracelets | | 0 | 1 |  |
| 239 | 1. A plain wedding band | | 0 | 1 |  |
| 240 | 1. Dangling earrings | | 0 | 1 |  |
| 241 | When should food handlers stay home from work? | |  |  |  |
| 242 | 1. When they are tired | | 0 | 1 |  |
| 243 | 1. When experiencing vomiting or diarrhoea | | 0 | 1 |  |
| 244 | 1. If they have a headache | | 0 | 1 |  |
| 245 | 1. If they are hungry | | 0 | 1 |  |
| 246 | What is the proper way to store raw meat in a refrigerator? | |  |  |  |
| 247 | 1. On the top shelf above ready-to-eat foods | | 0 | 1 |  |
| 248 | 1. On the same shelf as produce | | 0 | 1 |  |
| 249 | 1. On the bottom shelf to prevent dripping onto other foods | | 0 | 1 |  |
| 250 | 1. In the door compartment | | 0 | 1 |  |
| 251 | What should food handlers do after touching raw meat? | |  |  |  |
| 252 | 1. Continue working with clean food | | 0 | 1 |  |
| 253 | 1. Wipe hands on a towel | | 0 | 1 |  |
| 254 | 1. Wash hands thoroughly | | 0 | 1 |  |
| 255 | 1. Use hand sanitizer only | | 0 | 1 |  |
| 256 | How often should food-contact surfaces be cleaned and sanitized? | |  |  |  |
| 257 | 1. Once a day | | 0 | 1 |  |
| 258 | 1. Once a week | | 0 | 1 |  |
| 259 | 1. Every 4 hours during continuous use | | 0 | 1 |  |
| 260 | 1. Only when visibly dirty | | 0 | 1 |  |

**Part IV: Attitude of food handlers towards food safety practice**

| **S/N** | **Assessment questions** | **Strongly Agree**  **(n, %)** | **Agree**  **(n, %)** | **Neutral**  **(n, %)** | **Disagree**  **(n, %)** | **Strongly disagree**  **(n, %)** |
| --- | --- | --- | --- | --- | --- | --- |
| 1 | Personal hygiene is essential in preventing foodborne illnesses |  |  |  |  |  |
| 2 | Following food safety rules helps prevent foodborne illnesses |  |  |  |  |  |
| 3 | I feel responsible for maintaining food safety in my workplace |  |  |  |  |  |
| 4 | I always follow hand-washing procedures, even when I am busy |  |  |  |  |  |
| 5 | Wearing gloves while handling ready-to-eat food is necessary |  |  |  |  |  |
| 6 | I believe it is important to report co-workers who do not follow food safety rules |  |  |  |  |  |
| 7 | I feel comfortable reminding co-workers about food safety practices |  |  |  |  |  |
| 8 | If I feel slightly sick, I should still avoid handling food |  |  |  |  |  |
| 9 | Food safety training is beneficial for my job |  |  |  |  |  |
| 10 | I am committed to following food safety procedures at all times |  |  |  |  |  |
| 11 | Thoroughly washing chopping board prevent cross contamination |  |  |  |  |  |
| 12 | We have to ensure internal temperature of food are checked before use |  |  |  |  |  |

**Part V: Practice of food handlers towards food safety**

| **S/N** | **Questions** |
| --- | --- |
| 1 | Had purchased food inputs from the legal market or reputable suppliers? |
| 2 | Slaughter premises for all slaughtered animals? |
| 3 | Ensured the health status of all slaughtered animals by an authorized body? |
| 4 | Food handlers did not handle or process any food or meat when they had injuries to their hands? |
| 5 | Food handlers wear face masks or shields in public food establishments? |
| 6 | Food handlers reported washing their hands with soap for at least 20 s at key moments during work time? |
| 7 | Food handlers reported regularly trimming their nail and had seen the trimmed beard? |
| 8 | Food handlers wear a cap or head covering while selling meat, cooking, or serving food? |
| 9 | Food handlers wear a work cloth uniform or apron while working? |
| 10 | Food handlers had removed jewelers (hand and ring jewelers) while butchering, serving, or cooking food? |
| 11 | Food handlers use a thermometer to measure cooked food’s internal temperature? |
| 12 | Food establishments use refrigerator temperature monitors or tag? |
| 13 | Food handlers reported always cleaning and sanitizing areas of food establishments (kitchen, a place where meat is sold and stored)? |
| 14 | Food handlers reported double-checking the expiration dates of packed food input? |
| 15 | Food handlers never washed raw meats in the sink before cooking? |
| 16 | Food handlers reported discarding unsafe foods held at 40°F (9°C) for more than 2 h? |
| 17 | Food handlers reported food establishment managers monitor workers for food safety practices? |
| 18 | Public food establishments were supervised by authority bodies for their food safety practices? |

**Part VI: Qualitative data assessment questions**

**Questions for Key Informants:**

1. What are the key food safety practices that food handlers in Debre Birhan city are expected to follow?
2. How do you assess the level of knowledge and understanding of food safety among food handlers in the city?
3. What kind of training or orientation do food handlers receive on food safety, and how frequently is this training conducted?
4. Are there any common food safety violations you observe among food handlers in the food and drinking establishments in Debre Birhan?
5. What are the common practices among food handlers that might compromise food safety?
6. What do you think are the main challenges or barriers preventing food handlers from adhering to food safety practices?
7. Are there any financial or resource constraints that hinder food establishments from following proper food safety practices?
8. What role do community-based organizations or local media play in educating the public and food handlers about food safety?

**In-depth Interview Questions for Food Handlers:**

**Knowledge of Food Safety Practices:**

1. Can you describe the food safety practices you follow when preparing or serving food?
2. How were you trained or informed about food safety practices when you started working in this establishment?
3. How often do you receive food safety training or refreshers, and how useful do you find them?
4. What food safety measures do you take to prevent contamination or foodborne illnesses?
5. Are you aware of any common foodborne illnesses that can result from improper food handling? Can you give examples?
6. How do you deal with situations where you suspect that food may not be safe to serve to customers?
7. What measures are taken when foodborne illness outbreaks are suspected in your establishment?
8. What are the most common challenges you face while practicing food safety in your daily work?
9. Can you share any specific experiences where you faced difficulties in maintaining food safety, and how did you overcome them?
10. Do you believe the tools and equipment provided to you (such as gloves, thermometers, etc.) are adequate to maintain food safety? Why or why not?
11. How do you ensure that the food you handle stays safe from contamination during preparation, storage, and service?
